# Regularized Tensor Quantile Regression With Applications to Neuroimaging Data Analysis

**DOI:** 10.1002/sim.70582

**Published:** 2026-05-18

**Authors:** Matthew Pietrosanu, Dengdeng Yu, Ivan Mizera, Bei Jiang, Linglong Kong

**Affiliations:** ^1^ Mathematical & Statistical Sciences University of Alberta Edmonton Canada; ^2^ Management Science and Statistics University of Texas at San Antonio San Antonio Texas USA; ^3^ Probability and Mathematical Statistics Charles University Prague Czechia

**Keywords:** block relaxation, empirical processes, tensor regression, tensor regularization

## Abstract

This article proposes a regularized linear quantile regression model with a scalar response and tensor‐valued covariates. Our model uniquely regularizes the parameters of a low‐dimensional tensor effect decomposition through the tensor estimate rather than directly through the decomposition's parameters. We establish the computational and statistical properties of the proposed algorithm and estimators, both of which require separate treatment due to the quantile loss function. Simulation studies demonstrate the superiority of our model over existing tensor frameworks when traditional regression assumptions are violated. A real‐world neuroimaging analysis further highlights the interpretability benefits of our approach.

**Abbreviations**
CP: CANDECOMP/PARAFAC or canonical polyadic
mTBM: multivariate tensor‐based morphometry
SNR: signal‐to‐noise ratio

## 1 Introduction

Tensor‐valued data have grown ubiquitous in imaging, ecology, and chemometrics, among many other fields where large volumes of data are collected [1, 2, 3, 4] or where a functional response is sampled on a spatiotemporal grid [2, 5]. The analysis of tensor‐valued data is particularly important in neuroimaging and plays a crucial role in advancing the current understanding of human brain function and degeneration due to schizophrenia, Alzheimer's disease, and attention deficit hyperactivity disorder [6]. For example, tensor data can be derived from functional magnetic resonance imaging [3, 7] or diffusion tensor imaging [8, 9], two modalities commonly used to obtain dense measurements related to brain function.

Accommodating tensor‐valued data in regression poses unique statistical and computational challenges. As an extreme example, naively regressing a response onto a vectorization of an imaging tensor is unsatisfactory: this approach fails to address the high dimensionality of the original data and ignores intervoxel correlations. To address this, tensor decompositions [10], which are well established in the mathematical literature, have been applied for dimension reduction in statistical settings. Some existing methods for matrix and image analysis employ a two‐stage approach that estimates a decomposition in an unsupervised manner, conceptually similar to principal components analysis, and use the reduced feature set in a regression model [11, 12]. However, these reduced features are not guaranteed to be predictive of the response, and so two‐stage approaches are not ideal. Specific to tensor analysis, a generalized linear tensor regression model with regularization was previously developed in Zhou and Li [13] and Li et al. [14] to build on initial work in Zhou et al. [15] on modeling relationships between a scalar response and tensor covariates. As in other settings, however, the potential for violations of the standard assumptions of linear regression motivates the development of robust alternatives.

In many applications such as in the health sciences or finance, continuous responses of interest are often non‐normal, skewed, or corrupted with outliers [16]. Furthermore, data are often collected at various times or from different sources so that the traditional assumption of error homoskedasticity in linear regression might not be valid ([17], Ch. 2). These violations are all common in practice and support quantile regression [18] as an appealing alternative. Quantile regression models a conditional quantile of a response rather than its conditional mean and is robust against skewness, outliers, and error heteroskedasticity. In settings where the goal is to model extreme behavior, such as in clinical research for identifying trends in extreme cases or in finance for examining volatility, quantile regression can model the tails of a conditional response distribution. Thus, beyond its robustness properties, quantile regression offers a more comprehensive picture of the dependence structure between the response and model covariates.

In this paper, we develop a linear quantile regression framework for modeling the level‐τ quantile of a scalar response Y, conditional on both scalar‐ and tensor‐valued predictors. This model is

(1) $$Q_{\tau }(Y|x,Z)=\alpha_{\tau }+x^{⊤}\beta_{\tau }+\langle Z,\Gamma_{\tau }\rangle ,$$

where $Q_{\tau }$ denotes the level‐τ quantile of a scalar random variable for some fixed τ∈(0,1); $\alpha_{\tau }\in R$ is the model intercept; $x\in {\mathbb{R}}^{p_{0}}$ and $\beta_{\tau }\in {\mathbb{R}}^{p_{0}}$ are vectors of scalar covariates and their corresponding effects, respectively; and $Z\in {\mathbb{R}}^{p_{1}\times \cdots \times p_{q}}$ and $\Gamma_{\tau }\in {\mathbb{R}}^{p_{1}\times \cdots \times p_{q}}$ describe a tensor‐valued covariate and its corresponding effect, respectively. We define the inner product between two tensors in a standard way as $\langle Z,\Gamma_{\tau }\rangle ={\left(\text{vec}Z\right)}^{⊤}\text{vec}\Gamma_{\tau }$.

Statistical theory and computational approaches applicable to the generalized linear tensor model for mean regression are well‐established [14, 15]. However, analogous results for the quantile model in (1) are nontrivial and require separate treatment. Specifically, statistical properties of the standard quantile estimate of $\Gamma_{\tau }$ do not follow from those of the generalized linear tensor model as the quantile loss function does not correspond to a density function in the exponential family. Furthermore, the nondifferentiability of the quantile loss function necessitates further computational attention, as common first‐ and second‐order optimization methods are not directly applicable. This difficulty comes in addition to the unsurprising nonlinearity of the tensor model under typical tensor effect decompositions. Consequently, substantial developments are necessary to establish tensor quantile regression models.

This article's main contribution is a framework for incorporating tensor covariates into the robust class of quantile regression models. As shown in our simulation studies, the proposed framework is expected to be more robust to common violations of the assumptions in linear regression relative to existing tensor frameworks for mean regression. More specific contributions from the details of our framework are as follows.

First, unlike existing methods using an unsupervised decomposition in a two‐stage analysis [11, 12], our approach estimates a low‐rank Tucker decomposition [10, 19] that is optimally predictive of the specified response quantile. In effect, we perform tensor decomposition and model estimation simultaneously in the quantile setting. More‐recent developments in Ke et al. [20] and Li and Zhang [21] propose similar frameworks, but instead impose a CANDECOMP/PARAFAC (CP, also called canonical polyadic) structure on the regression coefficient. Since the assumption of Tucker or CP decomposition (the former technically generalizing the latter) is typically motivated by the problem at hand [10, 22], results for both are necessary.

Second, our framework emphasizes tensor effect interpretability and generalizability by indirectly regularizing tensor decomposition parameters through the estimated tensor effect ${\hat{\Gamma }}_{\tau }$: our approach differs from the regularized Tucker tensor models in Zhou and Li [13]; Zhou and He [23]; and Lu et al. [24], which instead directly penalize the components of the decomposition (i.e., the core tensor or factor matrices). The proposed framework consequently yields effect estimates that are more amenable to interpretation. In our numerical studies, we show that our regularized estimator mitigates noise and overfitting. Our neuroimaging analysis further shows that this estimator identifies spatial regions of positive and negative association with the response and has the potential to inform future neurological hypotheses and research.

Third, we establish important statistical properties of the proposed tensor effect estimator, including consistency and asymptotic normality, and convergence properties for the proposed algorithm. Our computational approach, which uses block relaxation to estimate tensor decompositions [25], theoretically accommodates a wide class of penalty terms that can be uniformly approximated by convex, differentiable functions: this includes lasso and its variants, as demonstrated in our numerical studies.

## 2 Tensor Quantile Regression

### 2.1 Model Estimation and Smoothing the Quantile Loss

Equation (1) defines the tensor quantile regression model with one tensor covariate. Extensions to multiple tensor‐variate predictors follow immediately and are discussed in Subsection 2.3. Since τ is generally set a priori, we suppress the subscript from here on to simplify notation. Model parameter estimates can be obtained by solving the optimization problem

(2) $$\underset{\alpha ,\beta ,\Gamma }{\arg\min}\;E[\rho_{\tau }(Y-\alpha -x^{⊤}\beta -\langle Z,\Gamma \rangle )],$$

where $\rho_{\tau }(u)=[\tau -1(u<0)]u$ is the quantile loss function defined for all real u.

This objective function in (2) immediately suggests a couple of difficulties. First, the problem is generally high dimensional, with $\prod_{j=1}^{q}p_{j}$ parameters from the tensor effect Γ alone: we address this with the Tucker decomposition introduced in Subsection 2.2. Second, the nondifferentiability of $\rho_{\tau }$ precludes the use of standard first‐ or second‐order optimization techniques such as gradient descent.

To address the latter issue, we introduce a differentiable approximation $\rho_{\tau ,\nu }$ of the quantile loss function with a smoothing parameter ν>0 and require that $\rho_{\tau ,\nu }$ converges uniformly to $\rho_{\tau }$ as ν→0. In this article, we take $\rho_{\tau ,\nu }$ as the generalized Huber function [26]

$$\begin{array}{l} H_{\tau ,\nu }(u)=\left\{\begin{array}{ll} u(\tau -1)-{\left(\tau -1\right)}^{2}\nu /2,\; & u<(\tau -1)\nu \\ u^{2}/(2\nu ),\; & (\tau -1)\nu \leq u<\tau \nu \\ u\tau -\tau^{2}\nu /2,\; & u\geq \tau \nu . \end{array}\right. \end{array}$$

### 2.2 Tucker Decomposition

To address the high dimensionality of the tensor effect, we assume a Tucker decomposition [10] of Γ with rank $\left(R_{1},\ldots ,R_{q}\right)$. Explicitly, this decomposition is

$$\Gamma =\overset{R_{1}}{\underset{r_{1}=1}{\sum }}\cdots \overset{R_{q}}{\underset{r_{q}=1}{\sum }}\lambda_{r_{1},\ldots ,r_{q}}\gamma_{r_{1}}^{\left(1\right)}\circ \cdots \circ \gamma_{r_{q}}^{\left(q\right)},$$

where ∘ denotes the usual vector outer product. From here on, this decomposition will be denoted by $\Gamma =[\Lambda |\Gamma^{\left(1\right)},\ldots ,\Gamma^{\left(q\right)}],$ where $\gamma_{k}^{\left(j\right)}$ is the kth column of the jth factor matrix $\Gamma^{\left(j\right)}\in {\mathbb{R}}^{p_{j}\times R_{j}}$ and $\lambda_{r_{1},\ldots ,r_{q}}$ is the $\left(r_{1},\ldots ,r_{q}\right)$th element of the core tensor $\Lambda \in {\mathbb{R}}^{R_{1}\times \cdots \times R_{q}}$.

Under a Tucker decomposition, the optimization problem (2) becomes

(3) $$\underset{\alpha ,\beta ,\Lambda ,\Gamma^{\left(1\right)},\ldots ,\Gamma^{\left(q\right)}}{\arg\min}\;E[\rho_{\tau }(Y-\alpha -x^{⊤}\beta -\langle Z,[\Lambda |\Gamma^{\left(1\right)},\ldots ,\Gamma^{\left(q\right)}]\rangle )],$$

but now the objective function is no longer convex in all its parameters. We will address this with a block‐relaxation algorithm that holds all but one of the q+2 parameter blocks in $\left\{(\alpha ,\beta ),(\Lambda ),(\Gamma^{\left(1\right)}),\ldots ,(\Gamma^{\left(q\right)})\right\}$ constant. We can rewrite the inner product of (3) as

$$\begin{array}{l} \langle Z,\Gamma \rangle =\left\langle {⨂}_{k=q}^{1}{\left(\Gamma^{\left(k\right)}\right)}^{⊤}\text{vec}Z,\text{vec}\Lambda \right\rangle =\left\langle Z_{\left[j\right]}{⨂}_{k=q,k\neq j}^{1}(\Gamma^{\left(k\right)})\Gamma_{\left[j\right]}^{⊤},\Lambda^{\left(j\right)}\right\rangle \end{array}$$

which makes it clear the model is linear when all but one of the parameter blocks are held constant. This observation motivates the following estimation algorithm.

### 2.3 Algorithm

Block‐relaxation algorithms are a general class of optimization techniques that subsume many well‐known methods such as majorize–minimization, alternating least squares, and expectation–maximization and come with established global and local convergence properties [27]. Cyclic variants optimize a scalar function ψ over a collection Ω of J parameter blocks by iterating sequential block updates of the form

$$\omega_{j}^{k+1}={\arg\min}_{\omega_{j}\in \Omega_{j}}\;\psi (\omega_{1}^{k+1},\ldots ,\omega_{j-1}^{k+1},\omega_{j},\omega_{j+1}^{k},\ldots ,\omega_{J}^{k}),$$

where superscripts denote the iteration in which an estimate was obtained.

In our setting, we use J=q+2 parameter blocks—one for the scalar parameters (α,β), core tensor Λ, and each factor matrix $\Gamma_{j}$. We assume a rule for determining a zero‐convergent sequence ${\left(\nu_{N}\right)}_{N}$ of positive smoothing parameters, with $\nu_{N+1}$ determined by $\nu_{N}$, the data, and current model estimates. We use $l_{N}$ to denote the loss function l in (3) but with $\rho_{\tau }$ replaced by the approximation $\rho_{\tau ,\nu_{N}}$. The rank $\left(R_{1},\ldots ,R_{q}\right)$ are model hyperparameters and can be tuned using the Bayesian information criterion, a cross‐validation criterion, or similar measures [28, 29, 30]. We propose Algorithm 1 for estimating the tensor quantile regression model.

In our implementation, we use a stopping criterion using a 0.01% relative change in the loss $l_{N}$ and perform optimization using gradient descent with a Barzilai–Borwein adaptive step size [31], which is known to perform well in high‐dimensional problems.

ALGORITHM 1 **Tucker tensor quantile regression.**

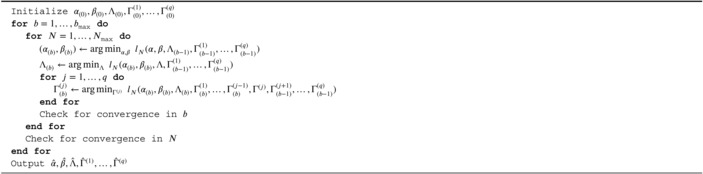

### 2.4 Regularization

Unlike previous works, we penalize the low‐rank structure imposed by the Tucker decomposition through the estimated tensor effect $\hat{\Gamma }$ rather than by penalizing the decomposition components directly. In the general setting with a differentiable scalar penalty function J(Γ), the optimization problem in (3) becomes

(4) $$\begin{array}{ll} & \underset{\alpha ,\beta ,\Lambda ,\Gamma^{\left(1\right)},\ldots ,\Gamma^{\left(q\right)}}{\arg\min}E\left[\rho_{\tau }(Y-\alpha -x^{⊤}\beta -\langle Z,[\Lambda |\Gamma^{\left(1\right)},\ldots ,\Gamma^{\left(q\right)}]\rangle )\right.\\ & \;+\left.\lambda J([\Lambda |\Gamma^{\left(1\right)},\ldots ,\Gamma^{\left(q\right)}])\right], \end{array}$$

where λ>0 is a penalization hyperparameter that can be selected using cross validation. For convenience, define $\Gamma_{j}^{⊗}={⨂}_{k=q,k\neq j}^{1}\Gamma^{\left(k\right)}$ and $\Gamma^{⊗}={⨂}_{k=q}^{1}\Gamma^{\left(k\right)}$. It follows that

$$\nabla_{\text{vec}\Gamma^{\left(j\right)}}J={\left[I_{p_{j}}⊗(\Gamma_{j}^{⊗}\Lambda_{\left[j\right]}^{⊤})\right]}^{⊤}\text{vec}{\left(\frac{\partial J}{\partial \Gamma }\right)}_{\left[j\right]}=\text{vec}[{\left(\frac{\partial J}{\partial \Gamma }\right)}_{\left[j\right]}\Gamma_{j}^{⊗}\Lambda_{\left[j\right]}^{⊤}]$$

and

$$\nabla_{\text{vec}\Lambda }J={\left(\frac{\partial \text{vec}\Gamma }{\partial \text{vec}\Lambda }\right)}^{⊤}\text{vec}\frac{\partial J}{\partial \Lambda }={\Gamma^{⊗}}^{⊤}\text{vec}\frac{\partial J}{\partial \Lambda }.$$

The above gradients are simple to incorporate into our implementation of Algorithm 1. The proposed algorithm also accommodates penalties that can be approximated by a sequence of differentiable functions $J_{\nu_{N}}$ that converge uniformly to J as N→∞. For example, the lasso penalty can be approximated by $\rho_{\tau ,\nu }$ with τ=0.5.

## 3 Model and Estimator Properties

### 3.1 Algorithm Convergence

We now establish the local and global convergence of the proposed algorithm for both a fixed $\nu_{N}$ and over the entire sequence ${\left(\nu_{N}\right)}_{N}$. Assume a monotonic, zero‐convergent sequence ${\left(\nu_{N}\right)}_{N}$ of positive smoothing parameters and fixed rank $\left(R_{1},\ldots ,R_{q}\right)$. Define the linear predictor $\eta =\alpha +x^{⊤}\beta +\langle Z,\Gamma \rangle$ and let θ denote the model parameters.

As data regularity conditions, we assume that the loss function $l_{N}$ is coercive, namely, that $\left\{\theta |l_{N}(\theta )\leq l_{N}(\theta_{\left(0\right)})\right\}$ is compact for any initial estimate $\theta_{\left(0\right)}$ of θ, and that the stationary points of $l_{N}$ are isolated. Previous works assume that each parameter block update is strictly convex, although this is clearly not the case for $\rho_{\tau ,\nu_{N}}$. Furthermore, the regularity condition assumed in Theorem 3.3 of Koenker and Bassett [18] that takes advantage of the nondifferentiability of $\rho_{\tau }$ no longer yields a unique solution interpolating some of the data due to the smoothness of $\rho_{\tau ,\nu_{N}}$. However, the continuity and convexity of $l_{N}$ and the assumed regularity conditions guarantee a unique solution in each update step. By applying the results of Fiorot and Huard [32], as discussed in Leeuw [27], and noting that the loss $l_{N}$ is strictly monotonic over parameter block updates and that the feasible set in each update is hemicontinuous in the fixed model parameters, global convergence follows immediately. This result still holds when considering a convex penalty function.

To establish local linear convergence, we follow the general discussion of Leeuw [27] that relies on the classical Ostrowski theorem [33]. This result requires that, at a fixed point $\theta^{*}$, the algorithmic update map is differentiable and that the spectral radius of the iteration Jacobian is strictly less than one. In the present setting, the algorithmic update map is differentiable as the composition of q+2 differentiable block update maps. Let M denote the iteration Jacobian, that is, the derivative matrix of the algorithmic map with respect to the model parameters. We require, more strongly, that at $\theta =\theta^{*}$, at least one of the residuals $y_{i}-\eta_{i}$ (i=1,…,n) is small enough so that the loss $l_{N}$ is strictly convex. Let L and D be strictly lower and diagonal block matrices, respectively, with the (i,j)th block (when nonzero) equal to $D_{i,j}l_{N}$, the derivative of $l_{N}$ with respect to the ith and jth parameter blocks. By strict convexity of its diagonal blocks, D is positive definite. It can then be shown that $M=-{\left(L+D\right)}^{-1}L^{⊤}$ and that M has a spectral radius that is strictly less than one. Consequently, local convergence holds and is linear by Ostrowski's theorem.

Moving beyond a fixed N, the more general result of Hjort and Pollard [34] proves that the algorithm iterates converge to the minimizer of the unsmoothed problem in (3). This result relies on the uniform convergence of the loss $l_{N}$ to the unsmoothed loss l that follows immediately from the uniform convergence of $\rho_{\tau ,\nu }$ to $\rho_{\nu }$. This still holds when a convex penalty function is considered, including the case where the penalty J is similarly approximated using a uniformly convergent sequence of convex, differentiable functions $J_{\nu_{N}}$.

### 3.2 Score, Information, and Identifiability

The problem of minimizing the objective function in (3) is equivalent to maximizing the log‐likelihood of independent and identically distributed asymmetric Laplace observations [35, 36] with the density function $f_{\tau }(y;\eta )=\tau (1-\tau )\exp\{-\rho_{\tau }(y-\eta )\}$. This distribution is not in the exponential class when η is unknown, so the following results do not follow from the generalized linear tensor model. Furthermore, the following results only apply to the unregularized setting, as an additional penalty term would violate the equivalence mentioned previously. One may consider estimator properties under a vanishing sequence of tuning parameters $\lambda =\lambda_{n}$, which still may be preserved under standard arguments for penalized M‐estimators (with convex or, as in our case, appropriately smoothed penalties) under the important assumption that $\lambda_{n}$ decays sufficiently quickly to zero. In what follows, we consider the unregularized estimator to characterize the intrinsic identifiability and asymptotic behavior of the underlying model without the additional bias introduced by an additional penalty. We treat regularization primarily as a device for finite‐sample stabilization and model selection in the numerical studies in the Section 2.

We derive the score function and information matrix when $\rho_{\tau }$ is replaced with $\rho_{\tau ,\nu }$. Define $f_{\tau ,\nu }(y)=C_{\tau ,\nu }\exp\{-\rho_{\tau ,\nu }(y-\eta )\}$ as the smoothed asymmetric Laplace density, where $C_{\tau ,\nu }>0$ is a normalizing constant. Despite the non‐differentiability of $f_{\tau }$, by uniform convergence, the score and information matrix for $f_{\tau ,\nu }$ will converge to that for $f_{\tau }$.

For notational simplicity, we ignore the intercept and scalar parts of the model, which are trivial to incorporate in theory. Define

$$J_{j}=\Pi_{j}\frac{\partial \text{vec}\Gamma_{\left[j\right]}}{\partial \text{vec}\Gamma^{\left(j\right)}}=\Pi_{j}[I_{p_{j}}⊗(\Gamma_{j}^{⊗}{\Lambda_{\left[j\right]}}^{⊤})],$$

where $\Pi_{j}$ is the row‐permutation matrix mapping $\text{vec}A_{\left[j\right]}$ to vecA for any tensor $A\in {\mathbb{R}}^{p_{1}\times \cdots \times p_{q}}$. The derivatives of η with respect to $\text{vec}\Gamma^{\left(j\right)}$ and vecΛ are, respectively, ${\left(\text{vec}Z\right)}^{⊤}J_{j}$ and ${\left(\text{vec}Z\right)}^{⊤}\Gamma^{⊗}$. The gradient of η with respect to the sequence of vectorized model parameters is then $\nabla_{\theta }\eta ={\left[\Gamma^{⊗}|J_{1}|\ldots |J_{q}\right]}^{⊤}\text{vec}Z$, which yields the score function

$$\nabla_{\theta }l_{N}=-\rho_{\tau ,\nu_{N}}^{\prime }(y-\eta ){\left[\Gamma^{⊗}|J_{1}|\ldots |J_{q}\right]}^{⊤}\text{vec}Z.$$

The expected Fisher information is as follows:

$$\begin{array}{ll} I_{N}(\theta ) & =𝔼[{\left(\rho_{\tau ,\nu_{N}}^{\prime }(y-\eta )\right)}^{2}]{\left[\Gamma^{⊗}|J_{1}|\ldots |J_{q}\right]}^{⊤}\\ & \;\text{vec}Z{\left(\text{vec}Z\right)}^{⊤}[\Gamma^{⊗}|J_{1}|\ldots |J_{q}]. \end{array}$$

By working in a restricted parameter space to resolve indeterminacy in the Tucker decomposition [10], it is clear that the assumptions required by Theorem 1 of Rothenberg [37] are met. We can conclude that the tensor quantile model is locally identifiable if and only $I_{N}(\theta )$ is nonsingular.

### 3.3 Consistency and Asymptotic Normality

To establish statistical properties of the proposed tensor effect estimator, we primarily employ properties of M‐estimators in empirical process theory [38]. Our arguments loosely follow those of Li et al. [14] and Zhou et al. [15].

Let ${\hat{\theta }}_{n}$ be the estimator of the true parameter values $\theta_{0}$ obtained from a sample of size n, and let ${\hat{\Gamma }}_{n}$ and $\Gamma_{0}$ be the corresponding tensor effect estimates. Let $M(\theta )=\mathrm{pr}\;l_{N}(Y|\theta )=\int_{R}l_{N}(y|\theta )f_{\tau ,\nu_{N}}(y|\theta )dy$ and $M_{n}(\theta )={\mathrm{pr}}_{n}\;l_{N}=n^{-1}\sum_{i=1}^{n}l_{N}(y_{i}|\theta )$, where ${\mathrm{pr}}_{n}$ denotes the empirical measure. By the strong law of large numbers, $M_{n}(\theta )$ converges almost surely to M(θ) as n→∞. The Cramer–Rao lower bound and local model identifiability are sufficient to guarantee that $\sup_{\theta :\;|\theta -\theta_{0}|\geq \varepsilon }M(\theta )<M(\theta_{0})$. The set {⟨Z,Γ⟩|Γ=Γ(θ)} is a Vapnik–C̆ervonekis class as a collection of polynomials of finite degree and is thus also Glivenko–Cantelli and Donsker. Furthermore, $\left\{l_{N}(y|\theta )|\theta \right\}$ is also Donsker under a restriction of the parameter space to a compact subset. With an appropriate envelope function that will depend on the smoothing approximation $\rho_{\tau ,\nu }$, this set is also pr‐Glivenko–Cantelli and pr‐Donsker. Thus, by the Glivenko–Cantelli Theorem ([38], Ch. 19), $\sup_{\theta }|M_{n}(\theta )-M(\theta )|$ converges in probability to zero. By Theorem 5.7 of van der Vaart [38], we can conclude that the sequence of estimators ${\hat{\theta }}_{n}$ converges in probability to $\theta_{0}$. Furthermore, by the open mapping theorem, ${\hat{\Gamma }}_{n}$ also converges in probability to $\Gamma_{0}$.

By Theorem 7.6 of van der Vaart [38], the continuous differentiability of $l_{N}$ and the existence and continuity of the elements of $I_{N}$ are sufficient to ensure the quadratic mean differentiability of the model estimated by minimizing the smoothed loss $l_{N}$. The asymptotic normality of ${\hat{\theta }}_{n}$ then follows from the above consistency result, quadratic mean differentiability, and Theorem 5.39 of van der Vaart [38]. In particular, for a neighborhood U of $\theta_{0}$, we take $\log p_{\theta }(y)$ and $\dot{l}(y)$ in the statement of the theorem to be $\log f_{\tau ,\nu }(y|\theta )$ and $\sup_{\theta \in U}|\nabla_{\theta }\log f_{\tau }(y)|$, respectively.

## 4 Numerical Studies

### 4.1 Simulation Studies

The following simulation studies examine the performance of the proposed tensor quantile model and the generalized linear tensor model under a setup similar to that in Li et al. [14]. We take q=2 with $p_{1}=p_{2}=64$ so that $\Gamma \in {\mathbb{R}}^{64\times 64}$ and $\beta ={\left(1,\ldots ,5\right)}^{⊤}\in {\mathbb{R}}^{5}$. Elements of $Z_{i}\in {\mathbb{R}}^{64\times 64}$ and $x_{i}\in {\mathbb{R}}^{5}$ (i=1,…,n) are generated independently from the standard normal distribution. The scalar response $Y_{i}$ is simulated as $Y_{i}=\eta_{i}+\varepsilon_{i}$, where $\eta_{i}=x_{i}^{⊤}\beta +\langle Z_{i},\Gamma \rangle$ and the $\varepsilon_{i}$s are independent and identically distributed. We consider normal, T, Cauchy, and $\chi^{2}$ distributions for $\varepsilon_{i}$: the parameters for each are set to achieve a specified signal‐to‐noise (SNR) ratio. We use the rank (R,R). We report mean absolute error on the test set as a measure of predictive performance and the root mean squared error of $\hat{\Gamma }$ as a measure of estimation accuracy. Given the difference in the objective function for the two frameworks, only the latter measure is truly meaningful for comparison. However, the two mean absolute error measures still speak to the robustness of each method to changes in the error distribution and other violations of standard regression assumptions. The following figures use a comparable color scale ranging from red (for large, negative values) to gray (zero) to blue (large, positive values).

**FIGURE 1:**
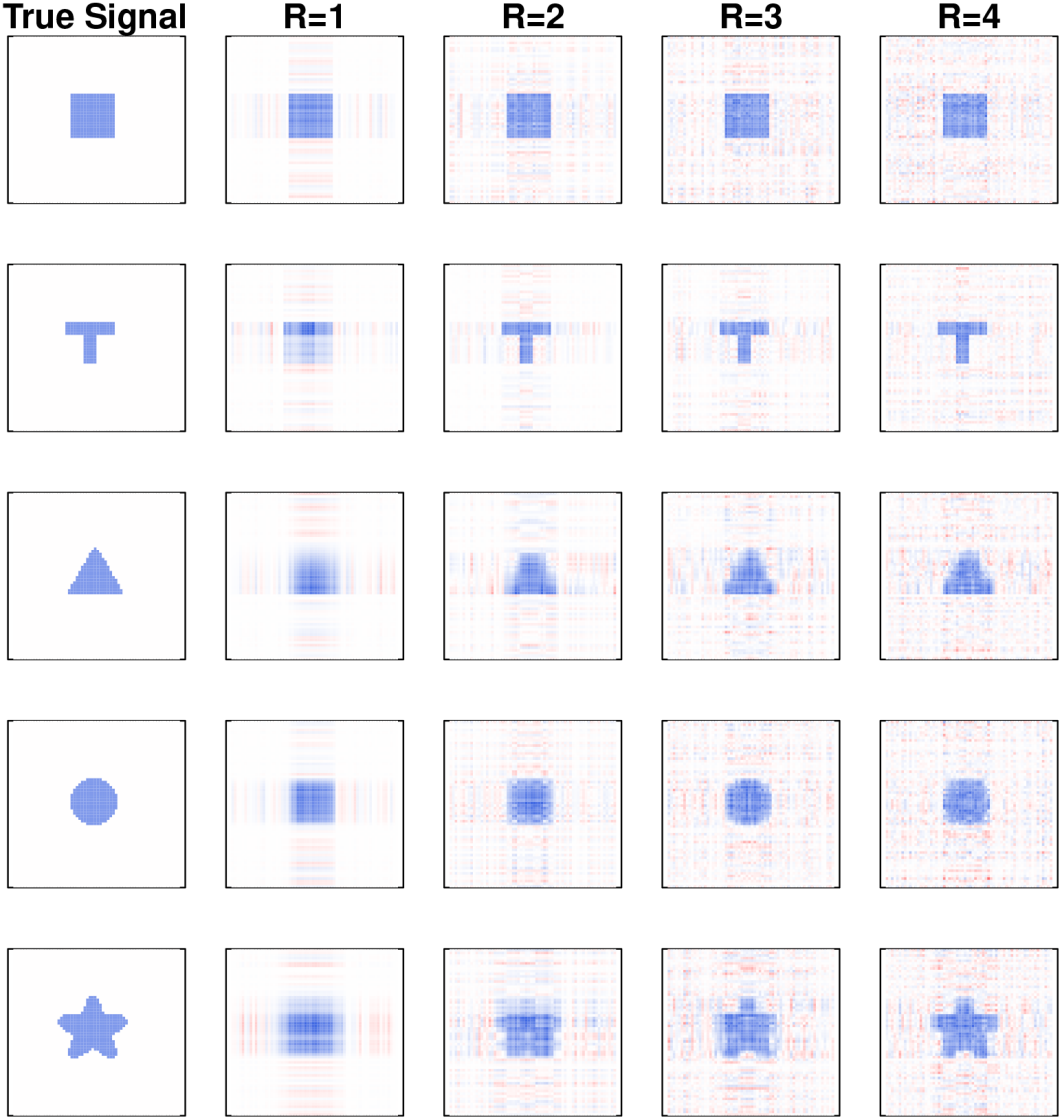
The true signal Γ (values in {0,1}) and tensor effect estimates obtained from the tensor quantile model with normal error (SNR=3) and n=2000.

**FIGURE 2:**
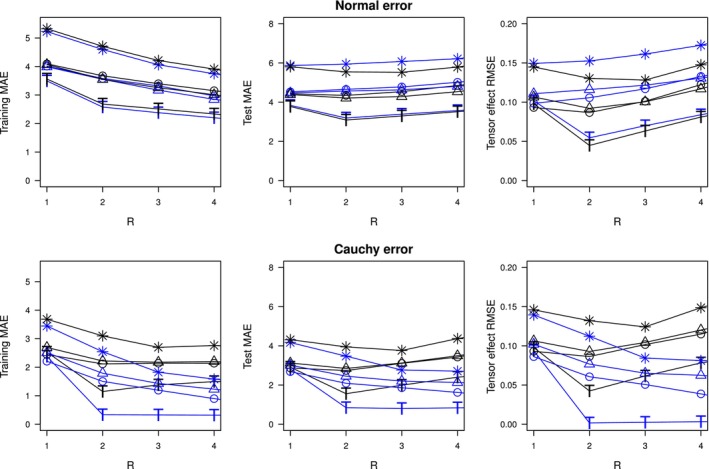
Model performance measures (blue and black for the linear quantile and generalized linear tensor model, respectively), averaged over five simulations with τ=0.5, SNR=3, and n=2000. Plot symbols identify the true signal.

Figure 1 shows five true tensor effects Γ with example estimates $\hat{\Gamma }$ from the proposed quantile model with normally distributed error. As shown in Figure 2 for normal and Cauchy error, training loss decreases and test error and estimator root mean squared error both exhibit a U‐shaped relationship with R. Visually, increasing R yields estimates $\hat{\Gamma }$ with greater background noise: this emphasizes the need for estimate regularization to mitigate overfitting. The proposed quantile model and the generalized linear tensor model show extremely similar performance in the setting with normally‐distributed error, but the former is clearly superior when the errors are Cauchy‐distributed. This result is expected, owing to the increased efficiency of quantile models when the error distribution is heavy tailed. Overall, the results suggest that the proposed tensor quantile model performs well under a variety of error distributions and is particularly well suited to heavy‐tailed errors. Tensor effect estimates behave as expected when varying other model parameters: performance degrades with a decreasing signal‐to‐noise ratio, deviations in the quantile level τ from 0.5, and decreasing sample size. Refer to the accompanying Supporting Informations for additional figures and tables with quantitative results.

**FIGURE 3:**
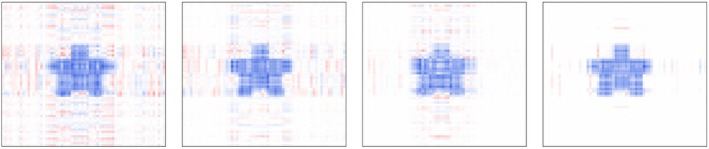
Lasso‐regularized tensor effect estimates for the star signal from the tensor quantile model with R=3, τ=0.5, normal error (SNR=3), and n=2000. From left to right, λ increases from 0 to 0.006.

To mitigate noise in unregularized tensor effect estimates, we apply an elementwise lasso penalty. In this particular simulation study, a penalty enforcing estimate smoothness does not seem necessary, unlike in the following neuroimaging analysis: Figure 3 shows estimates at varying values of λ, including the optimal λ determined by cross validation, for the star‐shaped signal. Visually, the estimates are less noisy and the true signal is easier to identify when regularization is applied.

### 4.2 Neuroimaging Data Analysis

We apply the proposed model to a real‐world dataset derived from the Alzheimer's Disease Neuroimaging Initiative [6] containing clinical and neuroimaging data from n=798 patients. Scalar covariates include gender, handedness, marital status, years of education, binary retirement status, and age. The scalar response is Mini‐Mental State Examination score, a clinical criterion used to monitor cognitive function and screen for neurodegenerative conditions such as dementia and Alzheimer's disease.

We use the same neuroimaging tensor data as Wang et al. [39] derived data from T1‐weighted magnetic resonance imaging scans of the hippocampus. This dataset includes surface‐based radial distance and three surface multivariate tensor‐based morphometry (mTBM) features, for a total of four tensor covariates. Wang et al. [39, 40] justify the use of surface‐based measures over traditional volumetric ones and notes increased statistical power to detect neurodegenerative diseases when these four tensor variables are considered together.

Each tensor feature was measured for each patient along the surface of the left and right hippocampi on the same 30,000‐point mesh in two variables. We reparameterized these measurements as an order‐3 tensor, as illustrated in the Supporting Informations. For computational convenience, we reduced the dataset by averaging each neuroimaging measure over discrete 2×2 partitions of the hippocampal surface, which resulted in one 2×75×50 tensor per variable per patient. We take $R_{1}=2$ and $R_{2}=R_{3}=R$, with R a hyperparameter to be determined, and τ=0.5.

**FIGURE 4:**
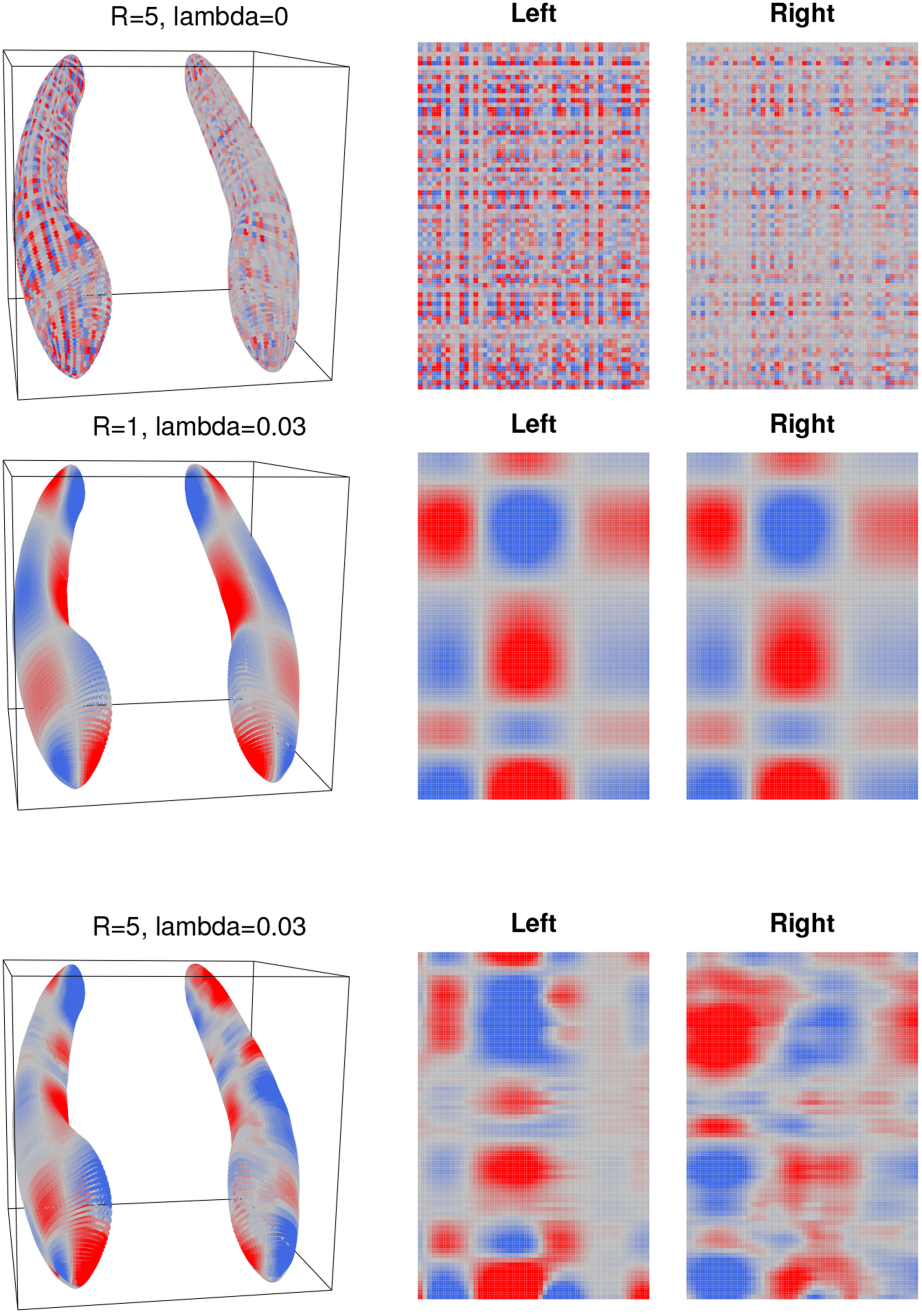
Tensor effect estimates for the first mTBM feature, visualized on the hippocampal surface and as an order‐3 tensor. Where regularization is present, λ is set to an optimal value as determined by cross‐validation. As before, the color scale ranges from red (for large, negative values) to gray (zero) to blue (large, positive values).

Unregularized tensor effect estimates show an extremely large amount of variability between adjacent voxels regardless of R and have no interpretive value. The unregularized estimate in Figure 4 with R=5 is visually similar to that with R=1 (shown in the Supporting Informations) and suggests a need to address this high variability across the hippocampal surface. We accomplish this with a fused lasso penalty J(Γ) equal to the sum of the absolute differences between elements of $\hat{\Gamma }$ corresponding to adjacent points on the hippocampal surface (with no penalty between the left and right hippocampi). Explicitly, $J(\hat{\Gamma })=\sum_{k=1}^{p_{1}}\sum_{i=1}^{p_{2}-1}\sum_{j=1}^{p_{3}}(|{\hat{\Gamma }}_{k,i,j}-{\hat{\Gamma }}_{k,i+1,j}|+|{\hat{\Gamma }}_{k,i,j}-{\hat{\Gamma }}_{k,i,j+1}|)$ with ${\hat{\Gamma }}_{k,i,p_{3}+1}={\hat{\Gamma }}_{k,i,1}$ for notational convenience.

Figure 4 displays tensor effect estimates at an optimal λ determined separately for R=1 and R=5. The effect of the fused lasso penalty is clearly visible in the smoother boundaries around identified regions of interest and the substantial reduction in estimated noise relative to the previous unregularized estimates. Regions of interest identified in the R=1 model seem to be restricted to smoothed rectangular regions influenced by the parameterization of the hippocampal surface, while the more complex model (with R=5) breaks from the rectangular parameterization. Regularized tensor effect estimates for all values of R identify similar patterns of positive and negative association between the tensor covariates and the response.

## 5 Discussion

Especially for imaging data, the ability to clearly identify regions of positive and negative association is extremely important, as these findings can inform future hypotheses and research. With this in mind, we argue that our approach to regularization can be more useful in practice than penalizing tensor decomposition parameters directly, as in Zhou et al. [15]; Zhou and Li [13], and Zhou and He [23], despite being less‐effective at further dimension reduction. The former reference briefly discusses the infeasibility of penalizing the estimated effect $\hat{\Gamma }$ without regard to tensor structure, but does not consider doing so through the assumed decomposition, as in this work. Our approach allows greater control over the type of regularization applied to $\hat{\Gamma }$: for example, the fused lasso penalty in our neuroimaging analysis would be much less meaningful if applied to the core tensor estimate $\hat{\Lambda }$ instead.

Instead of fixed‐order block updates, future work could update parameter blocks in some nondeterministic order via free‐steering block relaxation [25]. The latter is less‐developed in the literature and has fewer established theoretical guarantees. Other computational improvements might include [26, 41] residual‐dependent rules for updating ν or optimization via alternating direction method of multipliers, proximal gradient descent, or other paradigms.

While the theoretical properties and numerical studies in this work only consider convex penalties, nonconvex penalties such as smoothly clipped absolute deviation or the minimax penalty can easily be considered in practice. The usual care should be taken to avoid local minima, such as by considering a range of initial parameter estimates.

More generally, tensor regression models have the notable drawback of not truly accommodating functional data. Tensor regression does not estimate functional effects, does not explicitly account for the inherent smoothness of functional variables, and is not readily applicable to sparse settings where data is not sampled on a grid. We are currently developing a more general Bayesian functional tensor regression framework to this end.

## Author Contributions

M.P. and D.Y. were responsible for conceptualization and methodology. L.K. was responsible for data curation. M.P. was responsible for formal analysis, investigation, validation, visualization, and writing the original draft. I.M., B.J., and L.K. were responsible for supervision. All authors contributed to reviewing and editing of the manuscript.

## Funding

The authors acknowledge the financial support of the Natural Sciences and Engineering Research Council of Canada (NSERC) in this work. The research of Ivan Mizera was supported by the Czech Science Foundation, within its project GAČR No. 23–06461K, and by the NSERC of Canada.

## Conflicts of Interest

The authors declare no conflicts of interest.

## Supporting information




**Data S1**: Additional supporting information include additional visualizations and quantitative results for the simulation and neuroimaging analyses. These may be found in the online version of the article at the publisher's website.

## Data Availability

The data that support the findings of this study are openly available in ADNI at https://adni.loni.usc.edu/data‐samples/adni‐data/.
