# Supplementary material for: Regularized Tensor Quantile Regression With Applications to Neuroimaging Data Analysis
Source: Stat Med. 2026 May 18;45:e70582. doi: 10.1002/sim.70582 (PMC13183480; doi:10.1002/sim.70582)
Supplement: Supplementary file 1 — Data S1: Additional supporting information include additional visualizations and quantitative results for the simulation and neuroimaging analyses. These may be found in the online version of the article at the publisher's website. [file SIM-45-0-s001.pdf]

# Supplementary materials for "Regularized tensor quantile regression with applications to neuroimaging data analysis"

Matthew Pietrosanu*
Mathematical & Statistical Sciences, University of Alberta, Canada
and
Dengdeng Yu
Management Science and Statistics, University of Texas at San Antonio, U.S.A.
and
Ivan Mizera
Probability and Mathematical Statistics, Charles University, Czechia
Mathematical & Statistical Sciences, University of Alberta, Canada
and
Bei Jiang
Mathematical & Statistical Sciences, University of Alberta, Canada
and
Linglong Kong
Mathematical & Statistical Sciences, University of Alberta, Canada

February 17, 2026

*The authors acknowledge the financial support of the Natural Sciences and Engineering Research Council of Canada (NSERC) in this work. The research of Ivan Mizera was supported by The Czech Science Foundation, within its project GAČR No. 23–06461K, and by the NSERC of Canada. The authors acknowledge the financial support of the Natural Sciences and Engineering Research Council of Canada (NSERC) in this work. Data collection and sharing were funded by the Alzheimer's Disease Neuroimaging Initiative: for brevity, we refer to the initiative's homepage at http://adni.loni.usc.edu/ for a full list of funding sources.

# 1 Additional Simulation Study Results

Table 1: Estimator root mean square error for $\hat{\Gamma}$ under the tensor quantile regression model and the tensor general linear model for various true signals, error distributions, and tensor decomposition rank hyperparameter values. We use $\tau = 0.5$, a signal-to-noise ratio of 3, and $n = 2000$. Each value in the table is an average over five simulations.

| $R$ | Shape | Tensor quantile regression model | | | | Tensor general linear model | | | |
|---|---|---|---|---|---|---|---|---|---|
| | | Normal | Cauchy | T | $\chi^2$ | Normal | Cauchy | T | $\chi^2$ |
| 1 | Square | 0.052 | 0.001 | 0.040 | 0.046 | 0.041 | 0.042 | 0.039 | 0.040 |
| | T | 0.102 | 0.094 | 0.100 | 0.100 | 0.098 | 0.098 | 0.098 | 0.097 |
| | Triangle | 0.111 | 0.102 | 0.109 | 0.109 | 0.107 | 0.106 | 0.106 | 0.107 |
| | Circle | 0.099 | 0.086 | 0.096 | 0.096 | 0.093 | 0.093 | 0.093 | 0.093 |
| | Star | 0.149 | 0.139 | 0.148 | 0.149 | 0.145 | 0.146 | 0.145 | 0.144 |
| 2 | Square | 0.082 | 0.003 | 0.065 | 0.072 | 0.071 | 0.071 | 0.070 | 0.069 |
| | T | 0.055 | 0.002 | 0.045 | 0.049 | 0.045 | 0.042 | 0.043 | 0.043 |
| | Triangle | 0.116 | 0.077 | 0.102 | 0.099 | 0.092 | 0.093 | 0.091 | 0.092 |
| | Circle | 0.106 | 0.061 | 0.094 | 0.096 | 0.087 | 0.086 | 0.084 | 0.088 |
| | Star | 0.153 | 0.112 | 0.143 | 0.148 | 0.130 | 0.132 | 0.131 | 0.129 |
| 3 | Square | 0.099 | 0.004 | 0.086 | 0.089 | 0.092 | 0.092 | 0.094 | 0.094 |
| | T | 0.070 | 0.002 | 0.059 | 0.066 | 0.063 | 0.062 | 0.061 | 0.062 |
| | Triangle | 0.122 | 0.065 | 0.111 | 0.114 | 0.100 | 0.104 | 0.102 | 0.100 |
| | Circle | 0.117 | 0.051 | 0.110 | 0.106 | 0.101 | 0.102 | 0.099 | 0.101 |
| | Star | 0.161 | 0.084 | 0.141 | 0.139 | 0.128 | 0.124 | 0.125 | 0.129 |
| 4 | Square | 0.116 | 0.005 | 0.101 | 0.105 | 0.115 | 0.111 | 0.116 | 0.115 |
| | T | 0.084 | 0.003 | 0.074 | 0.075 | 0.081 | 0.078 | 0.078 | 0.077 |
| | Triangle | 0.131 | 0.062 | 0.124 | 0.119 | 0.117 | 0.120 | 0.118 | 0.116 |
| | Circle | 0.133 | 0.039 | 0.118 | 0.119 | 0.122 | 0.115 | 0.117 | 0.118 |
| | Star | 0.173 | 0.081 | 0.155 | 0.165 | 0.148 | 0.149 | 0.143 | 0.147 |
| 5 | Square | 0.131 | 0.006 | 0.115 | 0.120 | 0.134 | 0.127 | 0.135 | 0.136 |
| | T | 0.097 | 0.004 | 0.087 | 0.089 | 0.094 | 0.088 | 0.094 | 0.096 |
| | Triangle | 0.141 | 0.057 | 0.134 | 0.138 | 0.136 | 0.134 | 0.135 | 0.131 |
| | Circle | 0.145 | 0.025 | 0.137 | 0.136 | 0.139 | 0.136 | 0.139 | 0.140 |
| | Star | 0.191 | 0.078 | 0.166 | 0.179 | 0.172 | 0.168 | 0.168 | 0.173 |

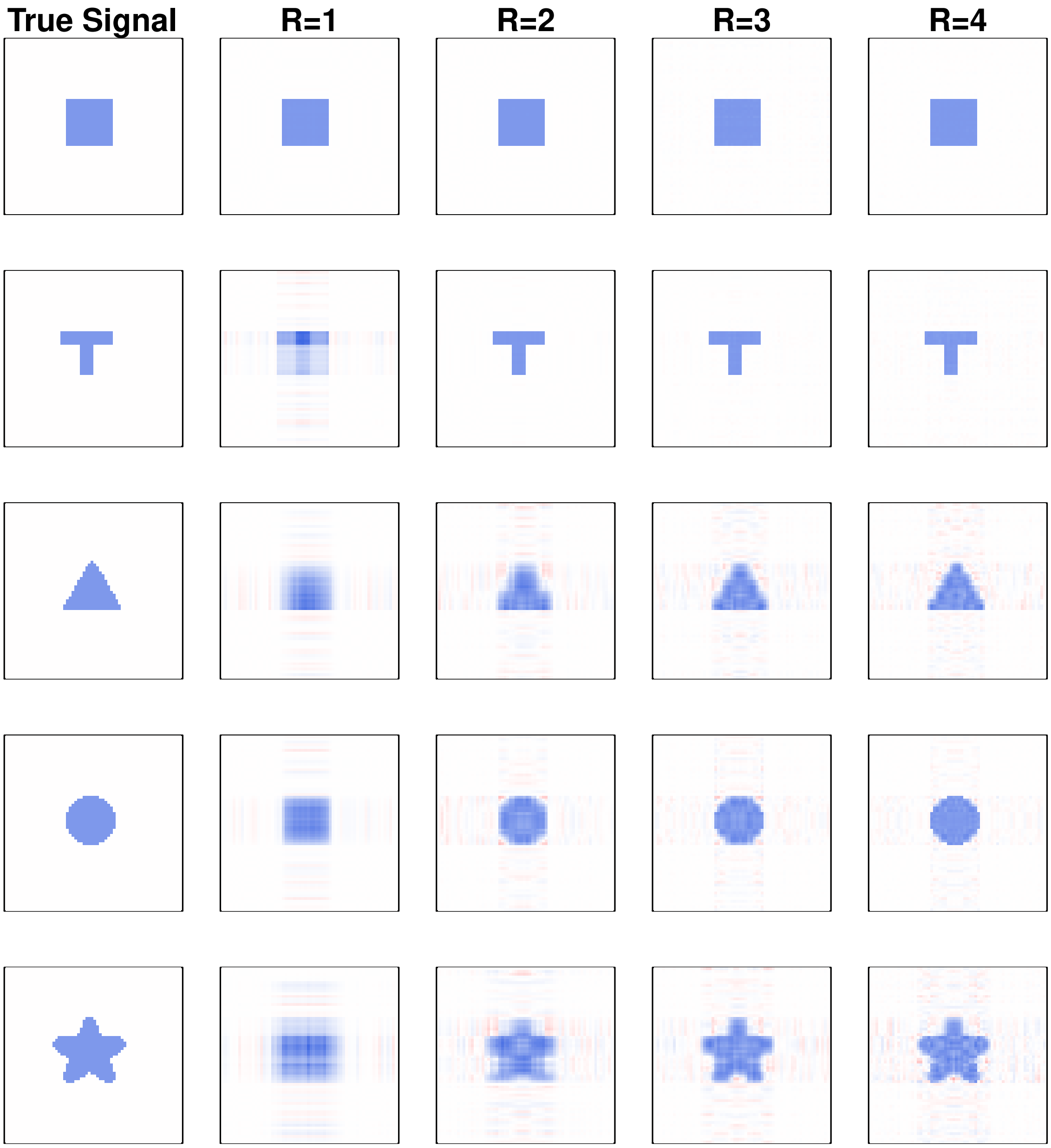


Figure 1: Examples of the true signal $\Gamma$ and tensor effect estimates $\hat{\Gamma}$ obtained from the tensor quantile model for five signals with $R = 1, \ldots, 4$, $\tau = 0.5$, a signal-to-noise ratio of 3, Cauchy-distributed error, and $n = 2000$. The colour of each pixel indicates the estimated effect size, which ranges from -1.5 (red) to 0 (white) to 1.5 (blue). The true signal is binary, i.e., each pixel has a value of either zero or one.

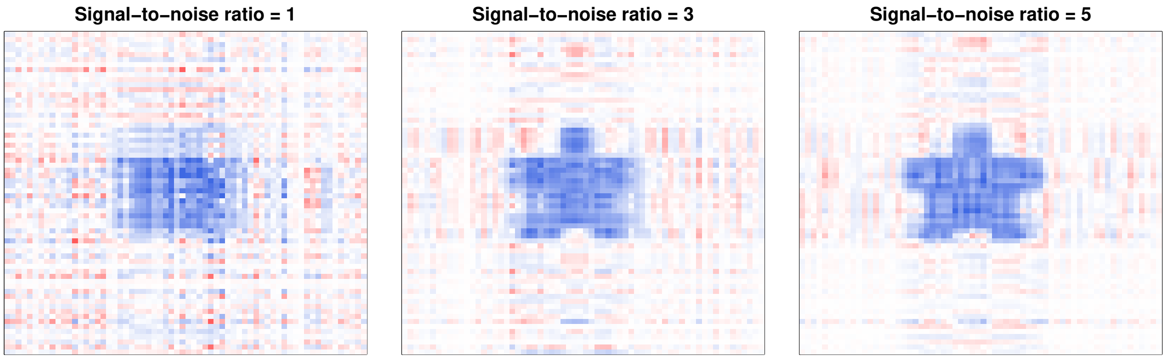


Figure 2: Examples of tensor effect estimates $\hat{\Gamma}$ of the star signal from the tensor quantile model for various signal-to-noise ratios with $R = 3$, $\tau = 0.5$, normally distributed error, and $n = 2000$. The colour of each pixel indicates the estimated effect size, which ranges from -1.5 (red) to 0 (white) to 1.5 (blue).

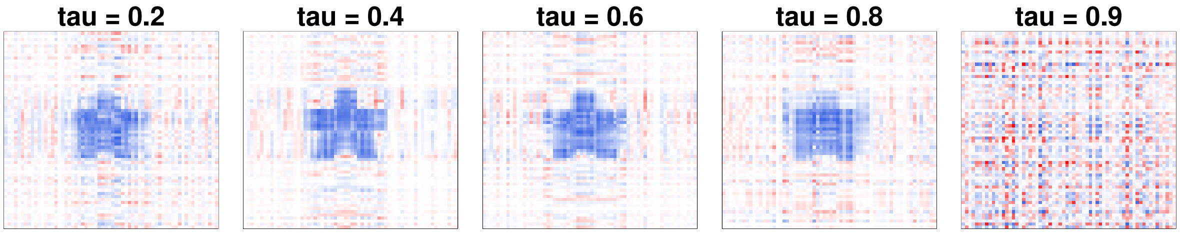


Figure 3: Examples of tensor effect estimates $\hat{\Gamma}$ of the star signal from the tensor quantile model at various quantile levels $\tau$ with $R = 3$, a signal-to-noise ratio of 3, normal error, and $n = 2000$. The colour of each pixel indicates the estimated effect size, which ranges from -1.5 (red) to 0 (white) to 1.5 (blue).

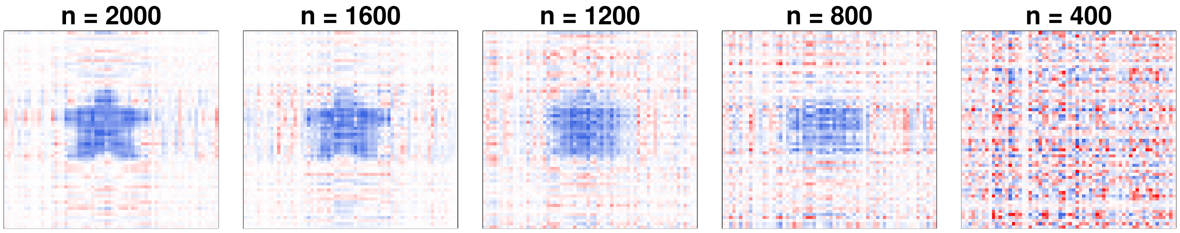


Figure 4: Examples of tensor effect estimates $\hat{\Gamma}$ of the star signal from the tensor quantile model at various sample sizes $n$ with $R = 3$, $\tau = 0.5$, a signal-to-noise ratio of 3, and normally distributed error. The colour of each pixel indicates the estimated effect size, which ranges from -1.5 (red) to 0 (white) to 1.5 (blue).

# 2 Additional Neuroimaging Data Analysis Results

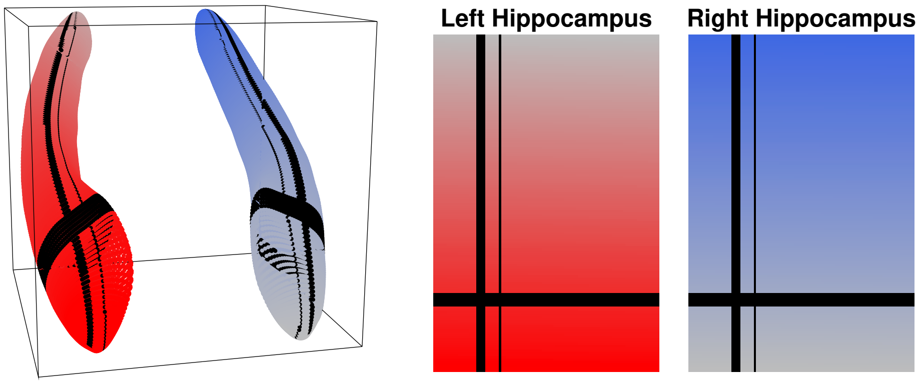


Figure 5: Visualization of the parameterization used in the main text of the hippocampal surface (left) to a $2 \times 150 \times 100$ tensor (right). Each subplot on the right represents one of the two $150 \times 100$ slices of the tensor observation matrix for a single neuroimaging measure. The colour gradient and black lines help illustrate the correspondence between the hippocampal surface and the tensor parameterization.

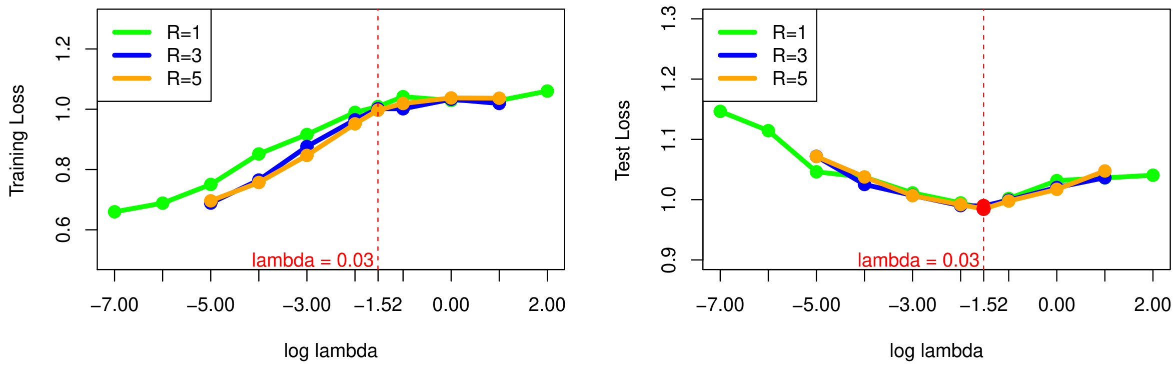


Figure 6: Training loss (left) and six-fold cross-validated test loss (right) as a function of the regularization parameter $\lambda$ for different values of $R$ in the neuroimaging analysis with fused-lasso regularization.

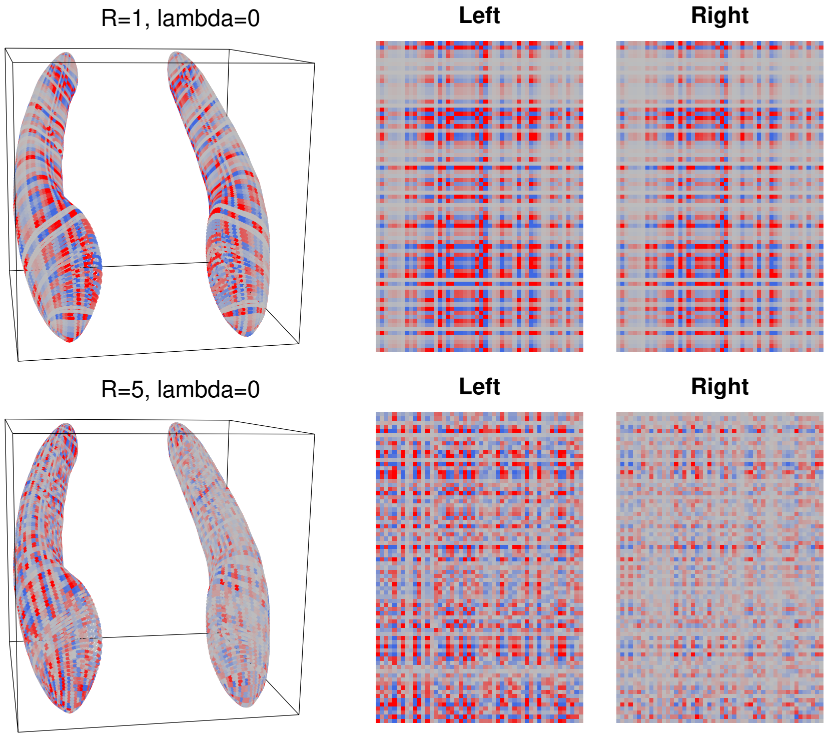


Figure 7: Comparison of the estimated effect of the first tensor-based morphometry variable, visualized as a tensor and on the hippocampal surface, for the unregularized $R = 1$ (top) and $R = 5$ (bottom) models fit to the full dataset.
